# Clinical research progress of ridaforolimus (AP23573, MK8668) over the past decade: a systemic review

**DOI:** 10.3389/fphar.2024.1173240

**Published:** 2024-03-22

**Authors:** Lumin Wang, Qining Qiu, Dawei Yang, Chang Cao, Yanqin Lu, Yulan Zeng, Weiwen Jiang, Yun Shen, Yanrong Ye

**Affiliations:** ^1^ Zhongshan Hospital (Xiamen), Fudan University, Xiamen, Fujian Province, China; ^2^ Zhongshan Hospital, Fudan University, Shanghai, China

**Keywords:** ridaforolimus, mTOR inhibitors, signaling pathway, drug combination, adverse events

## Abstract

Rapamycin, an established mTOR inhibitor in clinical practice, is widely recognized for its therapeutic efficacy. Ridaforolimus, a non-prodrug rapalog, offers improved aqueous solubility, stability, and affinity compared to rapamycin. In recent years, there has been a surge in clinical trials involving ridaforolimus. We searched PubMed for ridaforolimus over the past decade and selected clinical trials of ridaforolimus to make a summary of the research progress of ridaforolimus in clinical trials. The majority of these trials explored the application of ridaforolimus in treating various tumors, including endometrial cancer, ovarian cancer, prostate cancer, breast cancer, renal cell carcinoma, and other solid tumors. These trials employed diverse drug combinations, incorporating agents such as ponatinib, bicalutamide, dalotuzumab, MK-2206, MK-0752, and taxanes. The outcomes of these trials unveiled the diverse potential applications of ridaforolimus in disease treatment. Our review encompassed analyses of signaling pathways, ridaforolimus as a single therapeutic agent, its compatibility in combination with other drugs, and an assessment of adverse events (AEs). We conclude by recommending further research to advance our understanding of ridaforolimus’s clinical applications.

## Introduction

**FIGURE1:**
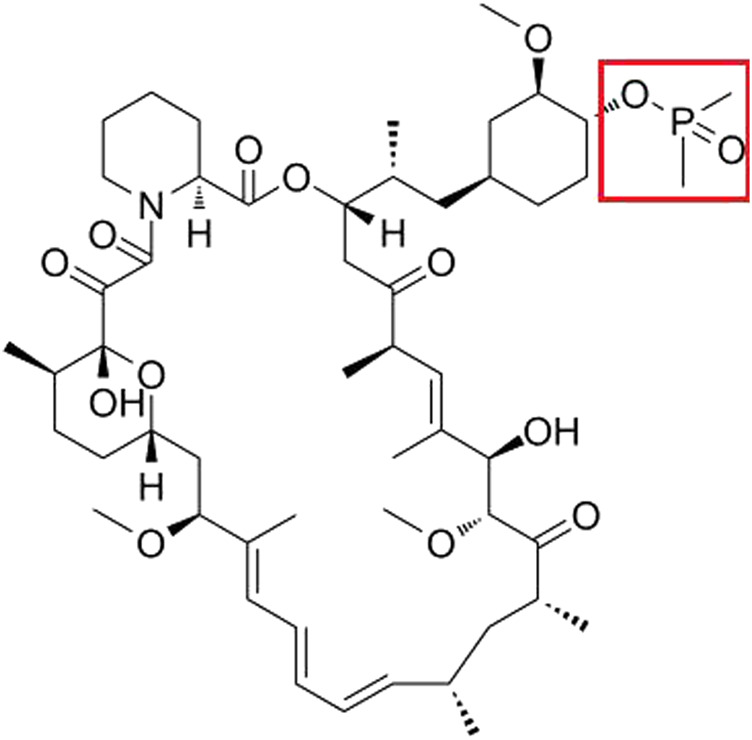
Structures of ridaforolimus.

The mammalian target of rapamycin (mTOR) is a critical receptor governing cell growth, metabolism, survival, and proliferation. Rapamycin, as the first-generation mTOR inhibitor, has demonstrated inhibitory effects on both the immune system and tumor proliferation (Bjornsti and Houghton, 2004; Guertin and Sabatini, 2005). Its mode of action involves the formation of a complex with FKBP12 within cells. However, rapamycin’s limited oral bioavailability and suboptimal solubility have constrained its clinical utility. Through computer-aided drug design (CADD), researchers identified the C40 site in the rapamycin structure as an ideal location for chemical modification, strategically distant from the binding sites of FKBP12 and mTOR. By introducing dimethyl phosphorylation at C40, a non-prodrug rapalog named ridaforolimus was developed, exhibiting superior aqueous solubility, stability, and affinity compared to rapamycin (Liu et al., 2009). A recent study showed that ridaforolimus could suppress immune responses and protect solid organ grafts in allotransplantation (Arash Boroumand Nasr et al., 2015). The structure of ridaforolimus is shown in Figure 1, where the red box represents the correction group of the C40 site of rapamycin.

Despite promising characteristics, ridaforolimus faced regulatory challenges. On 6 June 2012, the United States Food and Drug Administration (FDA) rejected its approval due to insufficient data. In response, we conducted a review of ridaforolimus research over the past decade, focusing on clinical trials of cancer therapy. Previous studies have consistently confirmed the safety and tolerability of ridaforolimus in patients. Notably, a single 40 mg dose of ridaforolimus was well tolerated in healthy adult males (Stroh et al., 2012a), and it exhibited consistent absorption rates regardless of whether it was administered with a light or high-fat breakfast. Furthermore, ridaforolimus did not prolong the corrected QT interval (QTc) in patients with advanced cancer (Lush et al., 2012). Our review of ridaforolimus research from 2012.01 to 2023.12 encompassed studies on the mechanism and clinical applications of ridaforolimus. The review is categorized into four key aspects, including signaling pathways that were affected by ridaforolimus, single-agent application of ridaforolimus in cancer therapy, drug combinations of ridaforolimus in cancer therapy, and the safety and adverse events (AEs) of ridaforolimus in cancer therapy.

## Signaling pathways that ridaforolimus affected

**FIGURE 2:**
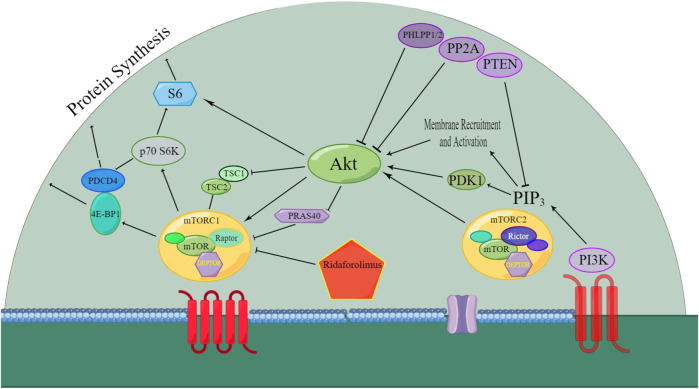
Overview of the signaling pathway that was affected by ridaforolimus. AKT: protein kinase B; mTORC1: mammalian target of rapamycin complex 1; mTORC2: mammalian target of rapamycin complex 2; 4E-BP1: eukaryotic translation initiation factor 4E-binding protein 1; PDK1: phosphoinositide-dependent kinase-1; PI3K: phosphatidylinositol-4,5-bisphosphate-3-kinase; PIP3: phosphatidylinositol (3,4,5)-trisphosphate (Liu et al., 2009; Stroh et al., 2012a; Arash Boroumand Nasr et al., 2015); PTEN: phosphatase and tensin homolog; S6K: ribosomal protein S6 kinase; DEPTOR: death domain-containing mTOR interacting protein; mTOR: mammalian target of rapamycin; Raptor: regulatory associated protein of mTOR; PDCD4: programmed cell death 4; TSC1: tuberous sclerosis complex 1; TSC2: tuberous sclerosis complex 2; PRAS40: proline-rich AKT substrate 40 kDa; PHLPP1/2: PH domain leucine-rich repeat protein phosphatase; PP2A: protein phosphatase 2A; and Rictor: rapamycin-insensitive companion of mTOR.

Figure 2 depicts the well-established signaling pathways of mTOR. The PI3K/AKT/mTOR pathway is of paramount significance in various cancers, such as renal cancer (Badoiu et al., 2023), gastric cancer (Fattahi et al., 2020), ovarian cancer (Ediriweera et al., 2019), non-small-cell lung cancer (Yip, 2015), acute myeloid leukemia (Nepstad et al., 2020), and endometrial cancer (Yunyun Li et al., 2016). Experimental validation has demonstrated the efficacy of the rapalog ridaforolimus in downregulating the PI3K/AKT/mTOR pathway (Nepstad et al., 2020). As a result, diverse cancer types exhibit characteristics such as cell cycle arrest, reduced cell size, and antiangiogenic effects (Berk et al., 2012).

The mTOR pathway’s activity is mediated through mTOR complex 1 (mTORC1) and mTOR complex 2 (mTORC2). Ridaforolimus specifically inhibits the mTORC1 signaling pathway by suppressing TRIM28 (tripartite motif containing 28) phosphorylation, hTERT (human telomerase reverse transcriptase) expression, and cell viability. It also reduces mutation sites in the hTERT promoters, specifically TRIM28 and TRIM24 (Agarwal et al., 2021).

DEPTOR blocks both mTORC1 and mTORC2 activities by directly interacting with mTOR, altering the DEPTOR/mTOR ratio upon the mTOR pathway inhibition. Studies suggest increased DEPTOR regulation as a positive prognostic marker in ovarian cancer, indicating the DEPTOR gene’s role in suppressing endometrioid ovarian cancer (Mita et al., 2013a).

Downstream markers of mTOR activity include phosphorylated 4E binding protein-1 (p-4E-BP1) and ribosomal protein S6 kinase-1 (p70S6K). Ridaforolimus has been shown to reduce the levels of p-4E-BP1 and pS6 in tumors or surrogate tissues, inhibiting their phosphorylation (Rivera et al., 2011).

In scorpion envenomation-induced pain, upregulation of the mTOR cascade and its downstream molecules, p70 S6K and eukaryotic initiation factor p-4E-BP1, occurs in the dorsal root ganglion (DRG). Inhibiting the mTOR cascade attenuates nociceptive behavior, highlighting its involvement in pain signaling (Jiang et al., 2013).

Phosphatase and tensin homolog (PTEN) mutations are rare in sarcoma cell lines, and even in cell lines expressing the wild-type PTEN gene, it fails to effectively inhibit PI3K signal transduction (Lim et al., 2016).

Recent *in vitro* studies reveal that ridaforolimus enhances cell susceptibility to severe acute respiratory syndrome coronavirus-2 (SARS-CoV-2) infection (Shi et al., 2022). This is achieved by countering the cell-intrinsic immune response through microautophagy, triggering the degradation of IFITM2 and IFITM3 and influencing susceptibility to SARS-CoV-2 infection. The transcription factor TFEB, associated with lysosome function through mTOR, emerges as a therapeutic target with the potential to activate a broad-spectrum antiviral response by modulating its expression.

In summary, the intricate modulation of the mTOR pathway by ridaforolimus presents diverse implications in cancer treatment, pain management, and antiviral strategies, emphasizing its multifaceted role in cellular processes. The multifaceted actions of ridaforolimus within the mTOR pathway present promising avenues for cancer treatment and offer intriguing insights into its potential role in viral infections, adding a layer of versatility to its therapeutic applications.

## Single-agent application of ridaforolimus in cancer therapy

The past decade has witnessed significant developments in single-agent ridaforolimus clinical trials, as summarized in Table 1. Notably, a pivotal study in 2013 evaluated the pharmacokinetics and safety profiles of ridaforolimus in Chinese populations, yielding favorable results. Chinese patients with refractory or advanced solid tumors exhibited good tolerance to a daily dosage of 40 mg ridaforolimus for five consecutive days, followed by two days off. The oral administration of ridaforolimus demonstrated a slow absorption with nonlinear blood pharmacokinetics similar to that observed in Japanese patients (Lian et al., 2013). Co-administration of drugs affecting stomach pH did not significantly impact ridaforolimus’s blood pharmacokinetics but did influence the dissolution of enteric-coated tablets. The findings indicated that ridaforolimus may stabilize the disease rather than causing tumor shrinkage in chemo-refractory tumor patients.

**TABLE 1:** Single-agent ridaforolimus clinical trials over the past decade.

Report time	Authors and refs.	Tumor type	Ridaforolimus dose and therapy (mg)	Research methods
2012	Lush et al. (2012)	Metastatic or locally advanced malignancy	Placebo on day 1 and a single 100-mg oral dose of ridaforolimus on day 2, 5 days later, 40 mg ridaforolimus QD × 5 days/week	Fixed-sequence, single-blind, placebo-controlled study
2012	Berk et al. (2012)	Implanted xenograft tumor	3-, 6.25-, 12.5-, 15-, 18.75-, and 28-mg QD ×5 every 2 weeks on a 4-week cycle	Open-label, single-center phase I, dose-escalation trial
2012	Mita et al. (2008)	Metastatic or unresectable solid tumors	40 mg QD × 5 days/week	A phase I/IIa, open-label, dose-escalation trial
2013	Gore et al. (2013)	Refractory solid tumors in pediatrics	3 + 3 dose escalation, dose levels of 8, 10, 13, and 16 mg/m^2^, 5 days/week	Multicenter, international, open-label, phase I study
2013	Lian et al. (2013)	Chinese advanced solid tumors	40 mg QD × 5 days/week	Open-label, single-center PK study
2013	Demetri et al. (2013)	Soft tissue and bone sarcomas	40 mg QD × 5 days/week	Phase III randomized double-blinded placebo-controlled multicenter international trial
2013	Colombo et al. (2013)	Endometrial cancer	12.5 mg intravenously once daily for 5 consecutive days every 2 weeks in a 4-week cycle	Open-label, single-arm, multicenter, phase 2 trial
2014	Tsoref et al. (2014)	Recurrent or metastatic endometrial cancer	40 mg QD × 5 days/week	A nonrandomized, open-label, multicenter, single-arm, phase II study
2015	Oza et al. (2015)	Advanced endometrial carcinoma	40 mg QD × 5 days/week	Open-label, multicenter, randomized, phase II trial
2016	Tsoref et al. (2014)	Pediatric patients with advanced solid tumors	22-, 28-, 30-mg/m^2^ QD × 5 days/week	Phase 1, multicenter, open-label study

QD: every day.

A phase I study investigating ridaforolimus in solid tumors was initiated in adults in 2008 (Mita et al., 2008), focusing on evaluating its pharmacokinetic and pharmacodynamic profiles. Similar trials were later carried out in children with refractory solid tumors, demonstrating consistent drug metabolism profiles between children and adults. Ridaforolimus was well tolerated and orally bioavailable in pediatric patients, although it led to grade 3 increases in alanine aminotransferase (ALT) (Andrew et al., 2016), and the maximum tolerated dose (MTD) was not established (Gore et al., 2013).

For women with advanced endometrial cancer, intravenous administration of 12.5 mg ridaforolimus once daily for five consecutive days, every two weeks, within a 4-week cycle, proved effective (Colombo et al., 2013). Standard oral ridaforolimus therapy extended progression-free survival (PFS) by 1.7 months (HR = 0.53) compared to the comparator arm (progestin or chemotherapy) (Oza et al., 2015). In 2013, a prospective, double-blinded, randomized phase III clinical study for soft tissue and sarcoma evaluated 40 mg oral ridaforolimus administered five days per week, revealing a 28% reduction in the risk of progression or death. Although standard ridaforolimus therapy led to a modest decrease in target tumor size according to independent review committee assessments and a slowing of disease progression (Demetri et al., 2013), the absolute increase in PFS was relatively small.

In glioma, ridaforolimus is associated with elevated cadherin-6 (CDH6) expression in high-grade glioma patient-derived tumor cells (Meng et al., 2022). This emerging association suggests potential implications for ridaforolimus in glioma treatment.

The past decade of single-agent ridaforolimus clinical trials has uncovered its diverse applications and tolerability profiles. From its promising effects in refractory tumors in Chinese populations to its efficacy in endometrial cancer and soft tissue/sarcoma, ridaforolimus showcases potential therapeutic benefits. Challenges, such as grade 3 ALT increases and limited PFS gains, underscore the need for further optimization and the exploration of combination therapies in future research endeavors.

## Drug combinations of ridaforolimus in cancer therapy

Significant developments in drug combinations of ridaforolimus in cancer therapy were summarize in Table 2. Ridaforolimus has been confirmed as a substrate for P-glycoprotein (P-gp) and is predominantly metabolized by the liver’s CYP3A enzyme, a key drug-metabolizing enzyme. Therefore, when ridaforolimus is combined with CYP3A inhibitors or inducers, it is crucial to consider the potential changes in its plasma concentrations. For instance, ketoconazole, a potent CYP3A4 inhibitor, significantly increased the AUC0–∞ (area under the curve) and Cmax (maximum concentration) of ridaforolimus by approximately 8.51- and 5.35-fold, respectively (Stroh et al., 2012b). Similarly, rifampicin, a CYP3A inducer, reduced the AUC of everolimus (a rapalog) by 63% (Effect of rifampin, 2002). Hence, during ridaforolimus treatment, strong CYP3A4 inducers or inhibitors should be avoided whenever possible. If unavoidable, dose adjustments for ridaforolimus may be necessary. On the other hand, ridaforolimus has a minimal impact on CYP3A activity, making it safe to use in conjunction with CYP3A substrates without requiring dose adjustments (Stroh et al., 2014).

**TABLE 2:** Drug combinations of ridaforolimus clinical trials over the past decade.

Report time	Authors and refs.	Tumor type	Ridaforolimus dose (mg) and therapy	Combination drugs and doses (mg)	Research methods
2012	Stroh et al. (2012b)	None	Part 1: ridaforolimus 40 mg d1, d14 + 600 mg rifampin daily for 21 days		Open-label, randomized, two-part study
			Part 2: ridaforolimus 5 mg d1, d2 + ketoconazole 400 mg daily for 14 days		
2012	Amato et al. (2012)	Castration-resistant prostate cancer	50 mg intravenous once weekly, at least 8 weeks	Taxane	Open-label phase II study
2012	Nemunaitis et al. (2013)	Advanced cancers	30 or 40 mg QD x 5 days/week	Bevacizumab 10 mg/kg Q2wk or 15 mg/kg Q3wk	A phase I, open-label, single-arm, cohort-based, dose-escalation trial
2013	Meulenbeld et al. (2013)	Asymptomatic, metastatic CRPC	30 mg QD x 5 days/week from day 8	Bicalutamide 50 mg/day from day 2	Prospective, open-label, international, multicenter safety lead-in trial
2014	Brana et al. (2014)	Advanced solid tumors	20 mg QD x 5 days/week	Dalotuzumab 10 mg/kg	Multicenter open-label phase I, parallel-arm study
2014	Di Cosimo et al. (2015)	Solid tumors	10–40 mg/day x 5 days/week	Dalotuzumab 10 mg/kg/week or 7.5 mg/kg/every other week	Phase I, international, multicenter, open-label, single-arm, nonrandomized trial
2014	Stroh et al. (2014)	Cancer	40 mg QD × 5 days/week	Midazolam, a single dose of 2 mg	Open-label, fixed-sequence, two-part study in 16 cancer patients
2015	Gupta et al. (2015)	Advanced malignancies; estrogen receptor-positive breast cancer or castration-resistant prostate cancer	3 + 3 dose escalation, 20 mg ridaforolimus + 90 mg MK-2206, 20 mg ridaforolimus + 135 mg MK-2206, 30 mg ridaforolimus +90 mg MK-2206, 30 mg ridaforolimus +135 mg MK-2206, 30 mg ridaforolimus +200 mg MK-2206, 40 mg ridaforolimus +135 mg MK-2206, 40 mg ridaforolimus +200 mg MK-2206, ridaforolimus QD × 5 days/week, MK-2206 once per week		Multicenter, international, open-label, nonrandomized two-part phase I study
2015	Zibelman et al. (2015)	Advanced renal cell carcinoma; solid tumors	3 + 3 dose-escalation design, 10 or 20 mg daily, 5 days/week	Vorinostat 100 mg daily	Single-institution, phase I investigator initiated study
2015	Piha-Paul et al. (2015)	Advanced solid tumors	20–30 mg, 5 days/week	MK-0752 (1800 mg once weekly)	An international, multicenter, open-label, nonrandomized, phase 1 study
2015	Seiler et al. (2015)	HER2^+^ trastuzumab-refractory metastatic breast cancer	40 mg QD x 5 days/week	Trastuzumab 4 mg/kg over 90 min + 2 mg/kg over 30 min (days 8, 15, and 22 of cycle 1 and days 1, 8, 15, and 22 of each subsequent cycle)	An open-label nonrandomized single-arm two-stage phase IIb trial
2016	Frappaz et al. (2016)	Pediatric patients with advanced solid tumors	28 mg/m^2^ QD x 5 days/week	Dalotuzumab 900, 1,200, and 1,500 mg/m^2^ intravenously Q3wk	A three-part, phase 1, multicenter, multinational, open-label, dose-escalation trial
2017	Baselga et al. (2017)	Estrogen receptor-positive breast cancer	30 mg QD x 5 days/week	Dalotuzumab 10 mg/kg/week or exemestane 25 mg/day	Randomized, multicenter, open-label phase-II study
2017	Rugo et al. (2017)	Advanced breast cancer	10 mg QD x 5 days/week	Dalotuzumab 10 mg/kg/week or exemestane 25 mg/day	Randomized, open-label, phase II trial
2017	Chon et al. (2017)	Solid tumor cancers	10–30 mg QD x 5 days/week, 3 + 3 design	Paclitaxel 175 mg/m^2^; carboplatin AUC 5–6 mg/mL/min	Phase I study

QD: every day; Q3wk: every 3 weeks; Q2wk: every 2 weeks.

A significant crosstalk exists between the PI3K/Akt/mTOR and androgen receptor signaling pathways. Bicalutamide, an androgen-receptor inhibitor, also affects CYP3A4 (Cockshott, 2004). Although combining anti-androgen agents with mTOR inhibitors was considered a reasonable approach, lower doses of ridaforolimus and bicalutamide were found ineffective for non-castration-resistant prostate cancer (CRPC) (Meulenbeld et al., 2013).

Vorinostat, a histone deacetylase inhibitor, can reduce AKT activation induced by ridaforolimus (Cranmer and Cranmer, 2014). Given mTOR’s dependence on AKT, adding vorinostat may overcome the resistance to mTOR inhibitors. A phase I study identified a tolerable dosing combination of ridaforolimus (20 mg daily) and vorinostat (100 mg daily) (Zibelman et al., 2015). Additionally, another AKT inhibitor, MK-2206, when combined with ridaforolimus, demonstrated promise in patients with breast carcinoma, both hormone-positive and -negative, showing favorable activity and tolerability (Gupta et al., 2015).

In endometrial cancer, the presence of activating mutations in fibroblast growth factor receptor 2 (FGFR2) and disruptions in the mTOR pathway have been identified as potential therapeutic targets. Studies have indicated that combining ridaforolimus with a low amount of ponatinib could achieve the ideal efficacy in endometrial carcinoma cells. This rational drug combination, targeting mutant-FGFR2 and mTOR-driven signaling pathways, holds therapeutic promise (Gozgit et al., 2013).

Notch signaling influences the PI3K pathway, which is crucial for cell proliferation, growth, and metabolism. Combining ridaforolimus and a Notch inhibitor, MK-0752, was anticipated to exhibit clinical activity. However, a relevant study in head and neck squamous cell carcinoma (HNSCC) patients encountered tolerability challenges, leading to the suspension of the study (Piha-Paul et al., 2015).

Dalotuzumab, an anti-insulin-like growth factor 1 receptor (IGF-1R) antibody, was evaluated in combination with ridaforolimus for various cancers, including solid tumors, endometrial cancer, and breast cancer. Phase I studies demonstrated that inhibiting the IGF-1R compensatory responses with mTOR inhibition could yield favorable clinical activity in advanced cancers, particularly in estrogen receptor (ER)-positive/high-proliferative breast carcinoma (Brana et al., 2014). While IGF-1R inhibition does not improve hormonal therapy in ER-positive breast carcinoma, likely due to compensatory IGF-2/IRA activity, *in vitro* studies showed synergy between dalotuzumab and ridaforolimus, potentially disrupting this feedback loop by targeting IGF-1R and IRS1 (a downstream target of IGF-1R) (Di Cosimo et al., 2015). Phase II studies in breast cancer indicated that the combination of ridaforolimus and dalotuzumab did exhibit antitumor activity, but it was associated with higher adverse events and showed no superior efficacy compared to exemestane in ER-positive breast carcinoma (Lamhamedi-Cherradi et al., 2016). Adding exemestane to the ridaforolimus and dalotuzumab combination improved the therapeutic outcomes in breast cancer. However, the triple therapy did not extend progression-free survival (PFS) beyond the double therapy in postmenopausal women with ER-positive and HER2-negative high-proliferation breast carcinoma (Rugo et al., 2017).

Metastatic breast carcinoma that is HER2+ and treated with trastuzumab can be challenging. Combining ridaforolimus with trastuzumab was found to offer benefits with good tolerability in patients with metastatic breast carcinoma (Frappaz et al., 2016). In recurrent or metastatic endometrial carcinoma, combining ridaforolimus with bevacizumab appeared tolerable, allowing for the full dose of ridaforolimus. However, the potential risk of drug-related bowel perforation should be considered (Tsoref et al., 2014). Nonetheless, further research comparing ridaforolimus with hormonal and chemotherapy regimens is needed to determine the optimal use of ridaforolimus in endometrial carcinoma.

Phase I studies combining oral ridaforolimus with paclitaxel and carboplatin have demonstrated anti-neoplastic activity without unexpected toxicity in patients (Chon et al., 2017). Phase II recommendations include a regimen of 30 mg ridaforolimus on days 1–5 and 8–12, 175 mg/m2 paclitaxel on day 1, and a single dose of AUC 5 mg/mL/min carboplatin, administered every 21 days per cycle. Similar phase II trials have been conducted, showing that when combined with taxane in prostate cancer treatment, ridaforolimus (50 mg, 5 days weekly) may maintain disease stability rather than achieving an objective response. This outcome has been consistent with the outcomes of previous studies, with researchers speculating that ridaforolimus may trigger mechanisms explaining the elevated prostate-specific antigen (PSA) levels in patients (Amato et al., 2012).

In conclusion, ridaforolimus exhibits versatile interactions and therapeutic potential across various cancers, but careful consideration of its combinations and their implications is essential for optimizing clinical outcomes. Future research should focus on refining these combinations for enhanced efficacy and reduced adverse events.

## Safety and AEs of ridaforolimus

In the assessment of AEs related to ridaforolimus, studies consistently utilized the “National Cancer Institute Common Terminology Criteria for Adverse Events.” Common AEs reported across various studies encompass stomatitis, infection, fatigue, thrombocytopenia, noninfectious pneumonitis, hyperglycemia, and rash.

Phase I trials of single-agent ridaforolimus highlighted mouth sores and rash as the most frequently observed AEs (Mita et al., 2008). In phase II studies, fatigue, stomatitis, and hypertriglyceridemia emerged as prevalent AEs, with stomatitis identified as a dose-limiting toxicity (Chawla et al., 2012). Additional AEs in phase II trials included anemia, diarrhea, nausea, vomiting, asthenia, and anorexia (Colombo et al., 2013; Oza et al., 2015). Pediatric populations exhibited recurring hematologic toxicity and electrolyte imbalances, albeit less frequently than in adults (Gore et al., 2013; Pearson et al., 2016).

Chinese patients, when administered ridaforolimus, demonstrated AEs and pharmacokinetic profiles akin to those of Caucasian and Japanese patients. However, proteinuria occurred more frequently in Chinese patients, with stomatitis and interstitial pneumonitis also being notable AEs in this population (Lian et al., 2013).

Combining ridaforolimus with ketoconazole introduced additional AEs, including mild and transient abdominal distension (Stroh et al., 2012b). Reduced ridaforolimus doses were used in combination with bicalutamide due to severe stomatitis, yet this combination led to grade-3 AEs such as hyperglycemia, mucosal inflammation, thrombocytopenia, and asthenia (Meulenbeld et al., 2013). Combining ridaforolimus with other agents, such as midazolam, an Akt inhibitor (MK-2206), and dalotuzumab, resulted in specific AEs, including rash and stomatitis, necessitating dose adjustments in some cases (Stroh et al., 2014; Gupta et al., 2015).

Combining ridaforolimus with dalotuzumab yielded AEs similar to previous reports, with stomatitis being evident at lower ridaforolimus dosages (20 or 10 mg), but fewer grade 3 stomatitis cases occurred with a 10 mg dosage compared to 20 mg doses (Baselga et al., 2017). Adding exemestane (25 mg/day) to the ridaforolimus and dalotuzumab therapy group resulted in less drug-related toxicities, suggesting the triple combination’s superiority over a reduced ridaforolimus dose in double combination (Rugo et al., 2017). Combining ridaforolimus with the cytotoxic chemotherapies paclitaxel and carboplatin led to more frequent hematologic AEs and similar non-hematologic AEs compared to previous single-agent ridaforolimus trials. The triple-therapy combination showed greater potential for bone marrow suppression, including neutropenia and thrombocytopenia, compared to double therapy (Chon et al., 2017). The common AEs of the ridaforolimus and vorinostat combination were oral mucositis (80.0%), fatigue (73.3%), anorexia (73.3%), thrombocytopenia (73.3%), hyperglycemia (60.0%), and anemia (60.0%) (Zibelman et al., 2015). Studies combining ridaforolimus with a Notch inhibitor, MK-0752, showed an approximately 32% incidence of stomatitis, diarrhea, and loss of appetite at the MTD, with rash not being a significant issue among common AEs for both agents (Piha-Paul et al., 2015).

Comparisons among mTOR inhibitors revealed that patients receiving ridaforolimus experienced a significantly higher frequency of grade 3–4 mTOR inhibitor-associated stomatitis (mIAS) compared to patients receiving temsirolimus and everolimus (Mita et al., 2013b). This discrepancy may be attributed to differences in dosing schedules and administration routes.

In summary, the diverse array of AEs associated with ridaforolimus underscores the importance of vigilant monitoring and tailored management strategies, particularly in the context of combination therapies. Understanding the distinct profiles of AEs across patient populations and in combination with various agents contributes to refining the clinical use of ridaforolimus.

## Discussion

Ridaforolimus, a promising mTOR inhibitor, has undergone extensive clinical evaluation, revealing a spectrum of AEs and demonstrating notable efficacy across various cancer types. Single-agent trials highlighted stomatitis, infection, fatigue, and thrombocytopenia as common AEs, while combination studies introduced additional considerations. Pharmacokinetic and safety assessments in diverse populations, including Chinese patients, underscored the drug’s general applicability, with unique observations such as increased proteinuria in the Chinese cohort. Combinations with ketoconazole, bicalutamide, and other agents showcased distinct AE profiles, necessitating careful dose adjustments.

Despite the encouraging results, limitations persist in the clinical use of ridaforolimus. AEs, including stomatitis, remain a challenge, emphasizing the need for tailored management strategies. Variability in AEs across populations and in combination studies poses complexities for clinicians. Moreover, the potential for dose-limiting toxicities, such as hyperglycemia and hematologic effects, warrants careful consideration in treatment planning. Additionally, the comparison with other mTOR inhibitors revealed variations in toxicity profiles, reflecting the importance of understanding distinct drug characteristics. Till now, there has been no comparative study of the anticancer effect of ridaforolimus and well-applied rapamycin, which would provide a more valuable reflection of the clinical application of ridaforolimus. A potential avenue for future clinical research could involve a comparative investigation into the respective roles of ridaforolimus and rapamycin in cancer treatment.

Looking ahead, ridaforolimus holds promise as a valuable therapeutic agent, particularly in combination strategies targeting various signaling pathways. Insights from studies combining ridaforolimus with vorinostat, Notch inhibitors, and cytotoxic chemotherapies contribute to the growing understanding of synergistic approaches. Further research exploring optimal dosing schedules, patient-specific factors influencing AEs, and mechanisms of action will refine the drug’s clinical utility. Ridaforolimus’s potential in endometrial cancer treatment, its role in overcoming resistance in certain combinations, and its interaction with the mTOR pathway in specific cancers offer exciting avenues for exploration.

## Conclusion

While challenges and limitations exist, ongoing advancements in the understanding of ridaforolimus pave the way for optimized therapeutic strategies. The drug’s multifaceted interactions, coupled with a nuanced approach to managing AEs, position ridaforolimus as a promising asset in the evolving landscape of cancer treatment.
